# The HEV Ventilator: at the interface between particle physics and biomedical engineering

**DOI:** 10.1098/rsos.211519

**Published:** 2022-03-16

**Authors:** Jan Buytaert, Paula Collins, Adam Abed Abud, Phil Allport, Antonio Pazos Álvarez, Kazuyoshi Akiba, Oscar Augusto de Aguiar Francisco, Aurelio Bay, Florian Bernard, Sophie Baron, Claudia Bertella, Josef X. Brunner, Themis Bowcock, Martine Buytaert-De Jode, Wiktor Byczynski, Ricardo De Carvalho, Victor Coco, Ruth Collins, Nikola Dikic, Nicolas Dousse, Bruce Dowd, Kārlis Dreimanis, Raphael Dumps, Paolo Durante, Walid Fadel, Stephen Farry, Antonio Fernàndez Prieto, Arturo Fernàndez Tèllez, Gordon Flynn, Vinicius Franco Lima, Raymond Frei, Abraham Gallas Torreira, Tonatiuh García Chàvez, Evangelos Gazis, Roberto Guida, Karol Hennessy, Andre Henriques, David Hutchcroft, Stefan Ilic, Artūrs Ivanovs, Aleksandar Jevtic, Emigdio Jimenez Dominguez, Christian Joram, Kacper Kapusniak, Edgar Lemos Cid, Jana Lindner, Rolf Lindner, M. Ivàn Martínez Hernàndez, Mirko Meboldt, Marko Milovanovic, Sylvain Mico, Johan Morant, Michel Morel, Georg Männel, Dónal Murray, Irina Nasteva, Niko Neufeld, Igor Neuhold, Francisco Pardo-Sobrino López, Eliseo Pèrez Trigo, Gonzalo Pichel Jallas, Edyta Pilorz, Lise Piquilloud, Xavier Pons, David Reiner, Hector David Règules Medel, Saul Rodríguez Ramírez, Mario Rodíguez Cahuantzi, Carl Roosens, Philipp Rostalski, Freek Sanders, Eric Saucet, Marianne Schmid Daners, Burkhard Schmidt, Patrick Schoettker, Rainer Schwemmer, Heinrich Schindler, Archana Sharma, Derick Sivakumaran, Christophe Sigaud, Vasilios Spitas, Nicola Steffen, Peter Svihra, Guillermo Tejeda Muñoz, Nikolaos Tachatos, Efstratios Tsolakis, Jan van Leemput, Laurence Vignaux, Francois Vasey, Hamish Woonton, Ken Wyllie

**Affiliations:** ^1^ European Organization for Nuclear Research, Espl. des Particules 1, 1211 Meyrin, Geneva, Switzerland; ^2^ Oliver Lodge Laboratory, University of Liverpool, Liverpool L69 7ZE, UK; ^3^ Particle Physics Group, School of Physics and Astronomy, University of Birmingham, Birmingham B15 2TT, UK; ^4^ Instituto Galego de Física de Altas Enerxías (IGFAE), Universidade de Santiago de Compostela, Santiago de Compostela, 15782 Galicia, Spain; ^5^ Nikhef National Institute for Subatomic Physics, Amsterdam 1098 XG, The Netherlands; ^6^ Institute of Physics, Ecole Polytechnique Fédérale de Lausanne (EPFL), CH-1015 Lausanne, Switzerland; ^7^ Neosim AG, CH-7000 Chur, Switzerland; ^8^ Tadeusz Kosciuszko Cracow University of Technology, 31-155 Cracow, Poland; ^9^ Hôpitaux Universitaires de Genève, 1205 Genève, Switzerland; ^10^ Department of Molecular Medicine, College of Veterinary Medicine, Cornell University, Ithaca, NY 14853, USA; ^11^ Prince of Wales Hospital, Randwick, New South Wales 2052, Australia; ^12^ University of New South Wales, Sydney, New South Wales 2052, Australia; ^13^ Applied Physics Laboratory, Faculty of Electronic Engineering, University of Nis˘, Aleksandra Medvedeva 14, Nis˘ 18000, Serbia; ^14^ University of Applied Sciences Offenburg, 77652 Offenburg, Baden-Wuerttemberg, Germany; ^15^ Deutsches Elektronen-Synchrotron (DESY), Platanenallee 6, 15738 Zeuthen, Germany; ^16^ Institute for Electrical Engineering in Medicine, University of Lübeck, 23562 Lübeck, Germany; ^17^ Facultad de Ciencias Físico Matemàticas, Benèmerita Universidad Autónoma de Puebla, Apartado Postal 165, 72000 Puebla, Pue., Mèxico; ^18^ Department of Physics and Astronomy, University of Manchester, Manchester M13 9PL, UK; ^19^ Universidade Federal do Rio de Janeiro (UFRJ), Rio de Janeiro 21941-972, Brazil; ^20^ Anesthesiology-Reanimation and Pain Therapeutics Service, Lucus Augusti University Hospital, 27003 Lugo, Spain; ^21^ Centre Hospitalier Universitaire Vaudois, Lausanne CH-1011, Switzerland; ^22^ John Curtin School of Medical Research, Canberra, Australian Capital Territory 2600, Australia; ^23^ GZA Hospitals, 2018 Antwerp, Belgium; ^24^ Cardio-Respiratory Units, Hôpital de La Tour, 1217 Meyrin, Switzerland; ^25^ National Technical University of Athens - NTUA, Zografou Campus, 15780 Athens, Greece; ^26^ Monash Health, Melbourne, Victoria 3168, Australia; ^27^ Dandenong Hospital, Melbourne, Victoria 3175, Australia; ^28^ Product Development Group Zurich, Department of Mechanical and Process Engineering, ETH Zurich, 8092 Zurich, Switzerland; ^29^ Centre of High-Energy Physics and Accelerator Technologies and Faculty of Computer Science and Information Technology, Riga Technical University, 1 Kalku Street, Riga LV-1658, Latvia; ^30^ Fraunhofer Research Institution for Individualized and Cell-based Medical Engineering (IMTE), 23562 Lübeck, Germany; ^31^ Adult Intensive Care Unit, University Hospital and University of Lausanne, Lausanne, Switzerland

**Keywords:** COVID-19, ventilation modes, triggering, oxygen enrichment

## Abstract

A high-quality, low-cost ventilator, dubbed HEV, has been developed by the particle physics community working together with biomedical engineers and physicians around the world. The HEV design is suitable for use both in and out of hospital intensive care units, provides a variety of modes and is capable of supporting spontaneous breathing and supplying oxygen-enriched air. An external air supply can be combined with the unit for use in situations where compressed air is not readily available. HEV supports remote training and post market surveillance via a Web interface and data logging to complement standard touch screen operation, making it suitable for a wide range of geographical deployment. The HEV design places emphasis on the ventilation performance, especially the quality and accuracy of the pressure curves, reactivity of the trigger, measurement of delivered volume and control of oxygen mixing, delivering a global performance which will be applicable to ventilator needs beyond the COVID-19 pandemic. This article describes the conceptual design and presents the prototype units together with a performance evaluation.

## 1. Introduction

Respiratory disease has a global impact as a major cause of morbidity, and the health challenges of the COVID-19 pandemic have significantly increased this burden. Medical ventilators are a key piece of equipment needed during COVID-19-related treatment, both in the acute phase, when invasive fully controlled ventilation is needed, and also in the sub-acute phase during the weaning from mechanical ventilation, which can last for an extended time period. The pandemic has brought into sharp focus the lack of reliable, high-quality ventilators in many regions of the world [1–4]. Chronic obstructive pulmonary disease (COPD) caused an estimated 5% of deaths worldwide in 2015 [5], the vast majority of which were in low- to middle-income countries, and pneumonia is globally the most common infectious cause of death [6–8]. The need for adequate respiratory equipment for treatment and management of such diseases will persist even as the COVID-19 pandemic wanes. Improving the situation is a challenge for the scientific community which crosses the boundaries of scientific expertise and can be addressed with new interdisciplinary collaborations.

A large number of proposals have been circulating since the start of the pandemic for devices which can be quickly manufactured cheaply and on large scale and may be open source [9,10]. The Illinois RapidVent [11] ventilator system is an example of a gas-powered pressure-switched design which can be rapidly produced using additive manufacturing at very low component cost; an approach also adopted in other projects [12–14]. Another popular design for many groups [15–20] is the motor-driven operation of resuscitation bags. These bags are readily available and can be found in all intensive care units (ICUs), where they are routinely used to manually ventilate patients, either intubated or wearing a mask. They consist of a self-inflating bag, a valve system to direct the air into the patient upon squeezing the bag and releasing the air from the lungs to atmosphere when letting the bag go. This arrangement is called BagValveMask, BVM. Such devices have the advantage of simplicity and are intended to address emergency needs. They are not intended to provide high-quality ventilation, including the delivery of high-flow pressure-controlled breaths with rapid rise times, measurement of delivered volume, precise synchronization with the patients’ breathing and control of oxygen of the delivered breaths. Low-quality solutions also require a much higher presence and attention of respiratory therapists, whereas the pandemic has exposed staff shortages. As has been recently highlighted [21], emergency devices often have limited functionality, monitoring and alarms, and are ill-suited for prolonged mechanical ventilation in patients with respiratory failure due to COVID-19.

Generally, two important aspects must be taken into account in the development of higher quality ventilators: suitability for spontaneous breathing support and the use of proper test devices for design verification. Spontaneous breathing support is extremely important in the ICU during the weaning phase. It requires proper triggering of a breath, delivery of variable gas flow at sometimes very high rates and patient-specific cycling to exhalation. Verification of the design cannot be done with simple bellows (called Test Lungs, for example SelfTest Lung, Dräger) because they lack specified compliance and resistance adjustments and do not provide spontaneous activity.

The HEV (high energy particle physics ventilator), first proposed here [22] is intended to address these needs and provide full functionality while being manufacturable at relatively low cost. The design is based on regulations and recommendations from the Medicines and Healthcare products Regulatory Agency (MHRA), EU, the Association for the Advancement of Medical Instrumentation (AAMI) and the World Heath Organization (WHO) [23–25]. HEV has been developed by a group of physicists and engineers affiliated to CERN^1^ and partner institutes, reinforced by an international advisory body of clinicians, with advice, collaboration and equipment provided from local hospitals. Compliance with ventilator design standards is inherently built into the design such that any eventual manufacturing will be possible according to these standards. High-quality ventilation provided by HEV includes the delivery of high-flow pressure-controlled breaths, measurement of delivered volume, precise synchronization with the patient's breathing and control of oxygen concentration of the delivered breaths. The HEV performance has been characterized with a fully specified lung simulator (TestChest, Organis GmbH, Landquart, Switzerland).

In this article, we demonstrate that by taking an idea familiar from the older traditional ventilators, i.e. an internal step-down pressure buffer, and combining it with improved modern components (valves, scroll pump, electronics, sensors) and control methodology, it is possible to provide ICU-level ventilation in a reliable, safe and relatively inexpensive way. The concept of a buffer with a lower pressure provides inherent safety and allows the design of the ventilator to be separated into two pneumatic stages with independent operation, giving greater freedom in the selection of components. On the input gas side, high-pressure small-bore valves can be selected. On the patient side, the valves do not need to be high pressure, so large internal diameters can be used, allowing excellent proportional-integral-derivative (PID) control sensitivity and fast reaction times. In addition, this architecture allows novel algorithms to be implemented, such as the independent volume calculation from the adiabatic pressure changes in the buffer. This compartmentalized design contrasts with an arrangement where all the pneumatic elements are inline and operate synchronously.

The design and controls of the HEV are based on concepts routinely used in the context of high energy physics research and use functionality which has been developed over decades for this field. The advantage of this cross-disciplinary approach and open collaboration is that the highest quality of ventilator construction can be expected, while being able to incorporate novel ideas during the development.

## 2. Overview of HEV functionality

Patients affected by COVID-19 face serious issues of lung damage, and the ventilatory equipment must be able to deliver pressure-controlled ventilation throughout situations of rapidly changing lung compliance as well as potential collapse and consolidation. In light of the prolonged recovery/weaning phases involved in COVID-19 critical care cases, the ventilator must also deliver pressure-assisted ventilation to be efficient for the ventilator weaning process. HEV^2^ delivers as basic modes pressure control—assist control (PC–A/C) and pressure control—assist control—pressure regulated volume controlled (PC–A/C-PRVC), pressure control—pressure supported ventilation (PC-PSV), and in addition continuous positive airway pressure (CPAP). The CPAP mode is included with the HEV modes in order to provide the widest range of support throughout COVID-19 treatment, and may be a crucial option for selection in low-resource settings [26]. In addition, HEV is capable of providing volume control modes. The proposed device can accurately supply target concentrations of oxygen enriched air and positive end-expiratory pressure (PEEP) is provided to support steady low positive pressure to the lungs to avoid alveolar collapse.

In all modes, an option is provided to measure the plateau pressure and intrinsic PEEP in order to provide clinical diagnoses and estimation of the patient static lung compliance or to detect AutoPEEP. This is achieved by allowing a manual operation of a pause time at the end of the inhalation phase during which the valves are occluded, in order to accurately measure plateau alveolar pressure at zero flow. The intrinsic PEEP at the end of the exhale phase can be measured in the same way during the pre-inhale state.

## 3. HEV conceptual design

### 3.1. Central pneumatic unit

**Figure 1.**
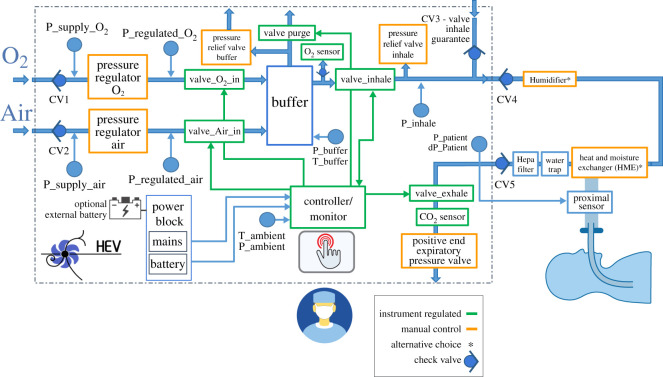
Conceptual design of the HEV ventilator.

The HEV design, shown conceptually in figure 1, is based around a central buffer which pneumatically decouples the ventilator circuit into two independently functioning parts, for filling the buffer and supplying gas to the patient. The air and oxygen are supplied separately and passively mixed inside the buffer, which is filled to a target pressure. Once this has been achieved, the buffer output valve is opened, initiating the respiratory cycle. The inhale valve is controlled by a PID (proportional–integral–derivative) controller, which allows a stable delivery of pressure and a fine tuning of the pressure rise time. After the inhalation phase finishes, the exhale valve is opened for exhalation and the buffer is refilled for the next breath cycle. For CPAP operation, the input valves to the buffer are kept open and the PID valve is regulated to supply a constant level of pressure. The PID algorithm is designed to ensure that the system is robust against fluctuations in flow or gas supply pressure.

The buffer concept presents many operational advantages. In general, the separation of the fill and exhale cycle into two separate circuits makes the design, control and component selection more straightforward, and allows less expensive components to be selected. The initial step-down of the pressure between the supply and the patient introduces safety and robustness, and makes precise pressure control on the patient side of the circuit more readily accessible. The buffer volume also avoids that the O_2_ and air delivery systems need to be able to handle the peak flow rates of up to 120 l min^−1^ needed in the inhale phase, and the patient air supply is protected. The mixing of the gases is provided inside the buffer, avoiding the need to purchase an external gas blender. In addition, the measurement of the O_2_ concentration, which can be done by spying on the static gas volume, is an inherently more precise measurement than measuring on a gas stream. Should the design need to be adapted to a more extreme (very hot or very cold) environment, thermal control of the gas in the buffer is straightforward. In addition, the delivered tidal volume, i.e. the amount of air which moves into the lungs during the respiratory cycle, can be calculated from the pressure drops in the buffer, providing a precious monitoring cross-check in addition to the standard tidal volume measurement from the proximal sensor, reinforcing the safety of the design.

### 3.2. Alternative air supply and accessories

The HEV collaboration has designed alternatives to the compressed air supply available in hospitals. The major requirement is that the system should be able to fill the HEV buffer in less than 1 s. Two different approaches are currently under consideration. The first consists of a small and transportable system, based on miniature turbine blowers installed in series, similar to the devices available for commercial ventilators. The air reaches thermal equilibrium with the environment via transport in a corrugated pipe before delivery to the ventilator via a high-efficiency particulate air (HEPA) filter. For increased independence from the hospital setting, an external battery can be included to power the turbine and the HEV together, with suitable batteries readily available.

**Figure 2.**
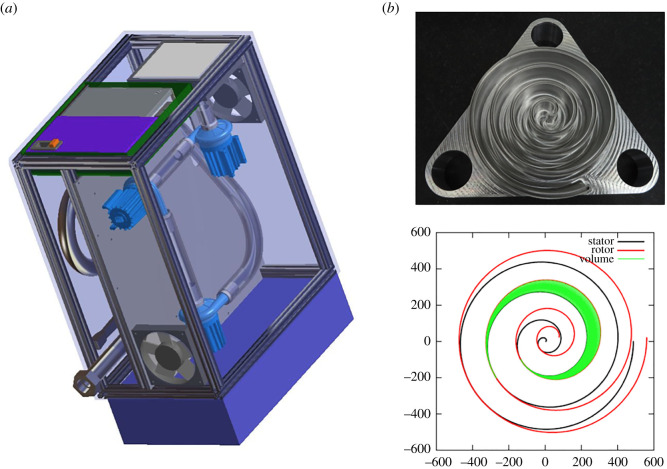
(*a*) Turbine system proposed as an alternative to the compressed air supply. The system is divided into two parts; the top part containing the turbines, the bottom containing the corrugated pipe, the thermal and pressure sensors and the outlet connector. Mounted on the left side of the box are the air filter, the power supply and the box containing the controller. The blue box on the right provides an enclosure for the optional battery system. (*b*) Photograph and snapshot of rotor stator profiles from the scroll pump design, which is being developed as a reliable and low-noise alternative to the turbine system.

A second approach has also been considered with the design of an air scroll pump oil-free compressor, based on prototypes already developed by members of the HEV collaboration [27]. Such a compressor has the advantage of being constructed with fewer parts, making them suitable for low resource settings, and a compact design with smooth and noiseless running, so well adapted for hospital ICU use. The design consists of scrolls with offset centres of rotation, which is optimized to have the lowest possible variation of the output delivered torque and volume. The unit can be constructed as an autonomous envelope for plug and play use to the HEV ventilator and integrated into the HEV control system and user interface. The designs of the two different systems are shown in figure 2; up to now, the turbine prototype on the left has been evaluated together with the HEV and shown to perform to specifications.

HEV has been tested with both a concentric tube geometry and a double limb circuit, and can be supplied with an adapter to use either method. The breathing circuit can also be equipped with humidifier or a heat and moisture exchanger (HME) filter by choice, which is necessary to protect the patient, who may be dehydrated, from the dry medical or ambient air.

All equipment that comes in contact with the patient needs to be either changed or disinfected and sterilized after every patient. There are multiple options to do this and currently autoclave cleaning is supported. The entire exhaust block may be easily dismounted and swapped with a spare block, so that the ventilator can continue to be used for the next patient while the block undergoes steam or autoclave sterilization.

### 3.3. Electrical design, control system and user interface

**Figure 3.**
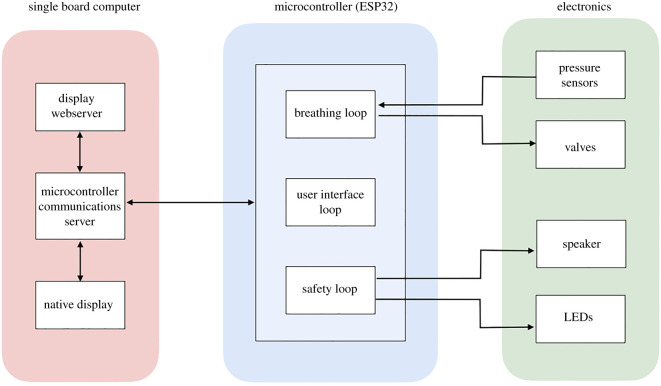
Diagram of the location of the different elements of hardware and software in the microcontroller, single board computer and circuit board.

The control software is implemented directly on an embedded controller, which receives signals from the sensors and valves, and fully controls the ventilator operation. For the HEV prototypes, an ESP32 microcontroller chip has been chosen for this function due to its high availability and low cost. Several medically compatible alternatives exist and the final choice will be made depending on local availability in different geographical locations. The microcontroller is connected to a single board computer which provides the user interface (UI) via a touchscreen, used for input and display of information. WiFi and Ethernet connection are also supplied via the single board computer. The microcontroller and single board computer are hosted on the principal motherboard, which is also equipped with connectors to access the input/output signals for valve control, sensing and other ancillary functions. The ESP32 uses pulse-width-modulation drive-integrated circuits on the mother board which transmits control signals to the valves, and the mother-board attenuates the signals from sensors before connection to the ESP32 for digitization. The motherboard also provides a number of embedded sensors for monitoring and debugging, light emitting diodes (LEDs) and a buzzer for user information and alarms, spare channels for additional valve and sensor connections, and connectors to allow the powering of fans and the touchscreen. A block diagram of the different elements of hardware and software is shown in figure 3.

The microcontroller software design consists of three distinct processing threads:

— A breathing loop responsible for operating the valves in a manner corresponding to the ventilator modes.
— A UI loop for relaying the current status and readings to the single board computer, and for accepting commands for setting modes and parameters.
— A safety loop which is responsible for raising alarms when patient or system readings deviate from acceptable limits.

For all loops, a finite state machine is employed to ensure operation is achieved in a repeatable and deterministic manner. The state machines operate semi-independently, with defined interfaces for information to pass between them. For example, this allows the safety loop to know both the current parameters set by the UI loop, and the current breathing loop ventilation mode. This allows each state machine to be tested individually by expressly checking the state response to each input at the interface.

All of the primary functions corresponding to the breathing function of the patient are controlled by the microcontroller. If the communications are interrupted to the single board computer, visual and audible alarms will trigger, but breathing and safety functions will continue while the connection is being re-established.

The HEV user interface has been designed in a way that ensures that the requirements according to the useability engineering process as found in IEC 60601-1 : 2005 + AMD1 : 2012, 12.2 and IEC 60601-1-6 : 2010 + AMD1 : 2013 can be satisfied. In particular, the design follows:

— Respect of regulatory guidelines for included quantities.
— Clear text, symbols and graphs which can be seen from the end of a hospital bed through personal protective equipment (PPE).
— Neutral colours for normal running; flashing indicators and messages when there are alarms.
— Screen locking/unlocking feature with a timeout to prevent accidental touchscreen presses.
— Confirmation required for all parameter changes (two parameter changes require two separate confirmations).
— Set and read back information clearly displayed.
— Simple navigation: no setting/parameter is more than two clicks away. Normal operation should be separated from calibration/expert testing, to maintain an uncluttered interface.
— Interface is touchscreen friendly: items are placed far enough apart to minimize accidental mis-clicks.
— Familiarity: designed to look familiar to clinicians, similar to already existing ventilator interfaces.
— Language selection is provided to increase regional adaptability.

Two user interfaces are provided: a native UI and a Web UI. The native UI runs on the touchscreen integrated into the ventilator unit. Remote access is provided via the Web UI, accessible via secure WiFi or Ethernet connection to the ventilator on computer screens or mobile devices. This also opens up the possibility of remote consulting and performance monitoring which can be very useful for training or patient management in remote settings. The Web interface can be configured so that full control, partial control, or no control is possible remotely. Access rights are configurable as required and can be disabled for reasons of security.

The native UI is automatically displayed on power-up of the device. At start-up, the mode is selected, with set parameters associated with that mode, which can be revisited at any time during operation. Indicators for the power source (mains or battery) are given on the UI, including residual charge indicators.

**Figure 4.**
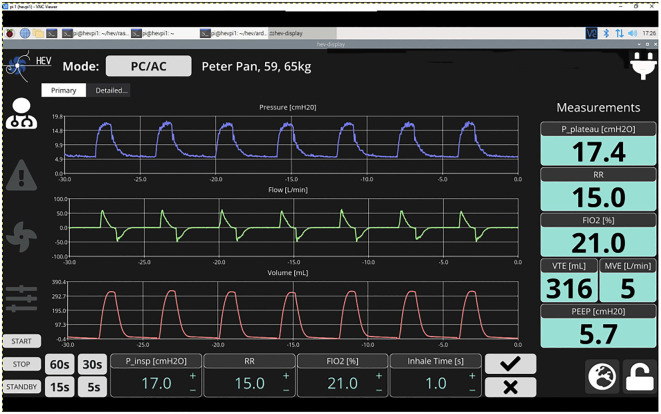
Example home screen display from the native user interface. Numbers and graphs are indicative.

A ‘home screen’ is shown for the current ventilation mode with the most important settings, parameters, waveforms and control buttons (figure 4). The native UI defaults to presenting the home screen after a period of inactivity by the user. A selection is provided for time ranges of the waveforms (i.e. showing the last 5, 15, 30 or 60 s). Historical data are recorded for up to 10 days. Encryption is an option for stored patient data. Settings (or target values) and measured values are clearly distinguishable in the UI.

The HEV Graphical User Interface is organized to respect the requirements listed in [24,25,28], in particular for the available information on the home screen, alarms, and monitored and controlled ventilating parameters. Alarms are implemented in a way such that the ISO 80601-2-12 : 2020(E) norms can be respected. All required alarms are present and include alarms related to the equipment operation (such as power or gas supply failure) and to the characteristics of the ventilation delivered to the patient (such as pressure, volume, fraction of inspired oxygen (FIO_2_) or respiratory rate) being above the maximum or below the minimum defined limits, and the presence of an obstruction or leakage. The alarms are displayed in an intuitive way at the top of the screen at all times. A dedicated alarm page shows a list of the last ten alarms, ordered by alarm priority, and current and historical alarms are easily distinguishable. It is possible to reset or silence alarms (for a period of time). The alarm on-screen visualization matches the three-level alarm indicator lights mounted on the unit: high (red), medium (flashing orange) and low (orange) priority. Finally, more technical details of the internal operation and calibration of the ventilator are provided on a separate ‘expert’ page.

HEV can run on AC power or an external battery, and includes an uninterruptable power supply (UPS) which takes over automatically if mains power is lost, allowing autonomous operation for up to 90 minutes.

## 4. HEV prototyping

Initially, three prototypes were constructed with an identical mechanical design in order to allow work in parallel on different aspects of the design implementation. In parallel, a fourth prototype was developed at the Galician Institute of High Energy Physics at the University of Santiago de Compostela (IGFAE/USC) with a twofold goal. Firstly, we wished to demonstrate that HEV could be reproduced easily in other places than CERN. Secondly, having successfully reproduced the design within a short timescale, we were able to launch additional development and contributions to the control software. In addition, this triggered discussions with local hospitals and physicians, further improving the design of the device.

**Figure 5.**
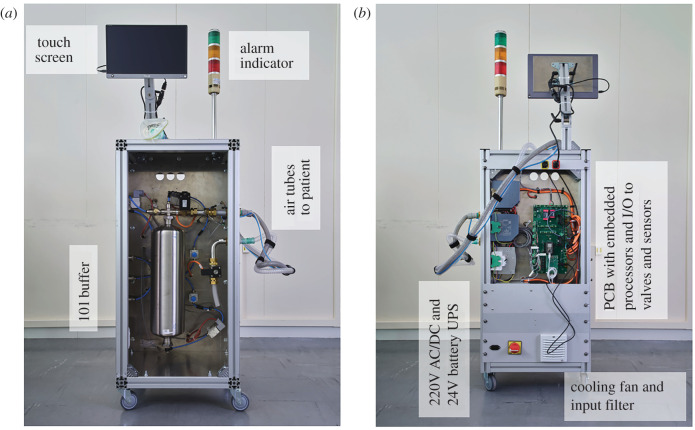
HEV prototype showing a front (*a*) and back (*b*) view.

Functionally, the prototype designs (figure 5) follow the concept described above. The resulting cabinet is mounted on wheels, can easily be moved by one person, is very stable, and provides a convenient surface to mount the display at head height. The cabinet is closed with doors, so that easy access for cleaning is possible. For safety, two compartments (front and back) provide complete separation between the pneumatic and electrical parts of the ventilator. The air tubes connect through a standard bulkhead thread connector on the outside, that make it easily replaceable to match hospital connection standards around the world.

### 4.1. Alternative mechanical designs

**Figure 6.**
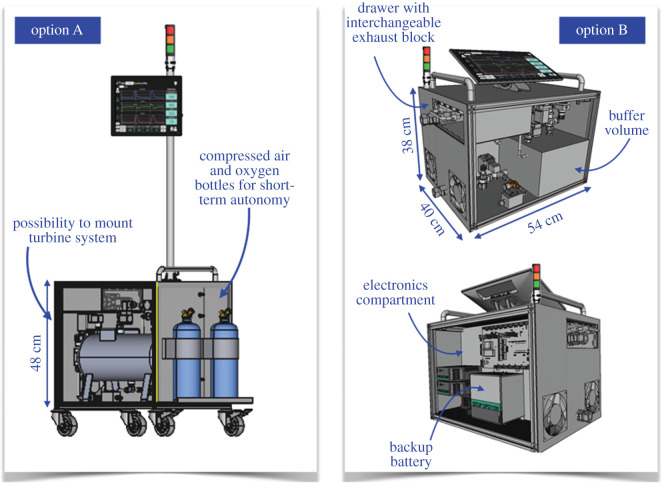
Potential alternative mechanical designs with an identical functionality to the HEV prototypes.

**Figure 7.**
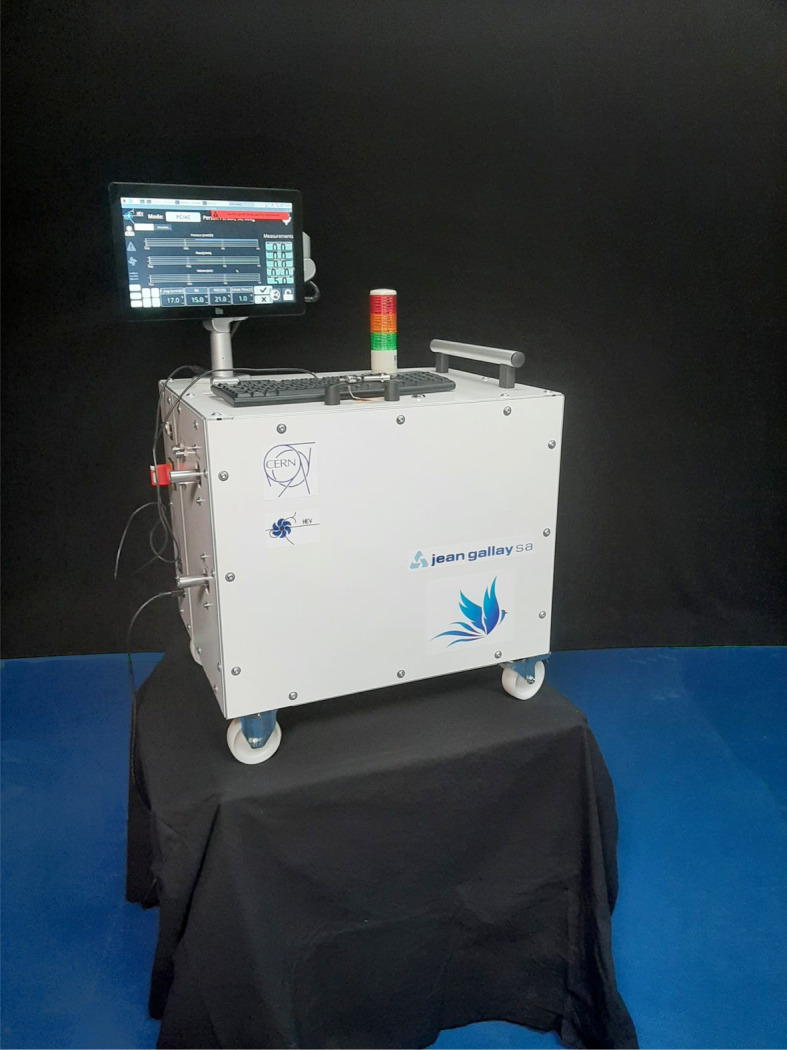
First of four prototypes to be produced in industry, to verify the HEV design parameters and optimize the form factor.

The prototypes were built with a deliberately large form factor to allow rapid development and exchange of parts. The final HEV ergonomics may look quite different depending on the requirements in the region of deployment and the accessories included. The HEV collaboration has provided two different alternative mechanical designs to which the HEV design could be adapted to fulfil different needs, which are illustrated in figure 6. Option A is more compact and can be mounted on wheels or a trolley. Space is provided to support oxygen and compressed air bottles, as well as the turbine system, so that the entire system and accessories can be provided as one integrated unit, which can be desirable for certain geographical locations. Option B is a still more compact and light version, for which the weight can be further reduced and the total dimensions are comparable to existing commercial ventilators. The touchscreen can be folded away for transport and the ventilator easily mounted on a trolley. Both options are identical in functionality to the HEV prototypes which have been built and tested. Initial prototypes are being constructed in industry to explore the potential of the new mechanical designs. So far, four prototypes have been produced, with identical functionality to the initial prototypes described in this paper, but with smaller form factors, and a final target weight of 24 kg. Figure 7 shows the first of these prototypes to be produced.

## 5. Prototype test results

**Figure 8.**
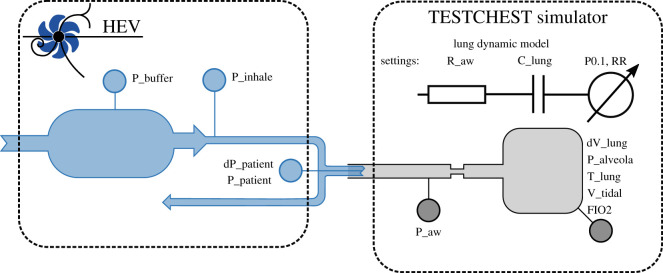
Ventilator test stand.

The set-up used to evaluate the HEV performance is equivalent to that specified by MHRA [28] and ISO 80601-2-12 Fig. 201.102 [29] and illustrated in figure 8. The HEV is tested using the TestChest (Organis GmbH, Landquart, Switzerland) which is a fully specified test lung whose features include remote controllable lung mechanics such as resistance, compliance and leakage, spontaneous breathing with predefined patterns of respiratory rate and activity, and oxygen concentration measurement. The physiological model of TestChest is described in [30]. In setting the patient profiles the MHRA guidance was followed, with nominal patient settings with a compliance of 50 ml mbar^−1^ and airway resistance of 5 mbar l^−1^ s^−1^, and then varying the settings between 10–100 ml mbar^−1^ and 5–50 mbar l^−1^ s ^−1^, respectively, to investigate more obstructive and restrictive patient models. In the tests which follow the settings, respiratory rates and PEEP levels are indicated. For the inhalation trigger testing different levels of patient effort were investigated, as detailed below.

### 5.1. Target pressure performance

**Figure 9.**
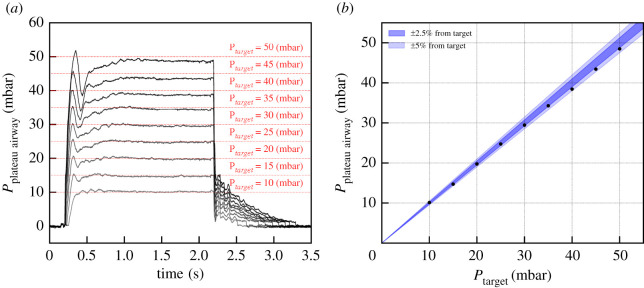
(*a*) Pressurization of a 20 cm H_2_O ml^−1^ compliance, 5 cm H_2_O l^−1^ s^−1^ resistance patient with various target pressures. The inhalation time is set to 1.5 s, and is followed by a pause of 0.5 s. (*b*) Deviation of the inhale pressure from the target as computed during the pause.

As discussed in §3.1, when the inhale starts, the proportional inhale valve opens in a controlled way in order to reach the target pressure in a given time. This is done through a PID controller that monitors the pressure measurement as input for the inhale valve opening control. As illustrated in figure 9, any pressure level within the setting range can be reached and maintained with an uncertainty below 3%.

The performance was checked using the leak settings available with TestChest and even with the highest leak setting, no difference in the pressure profile is visible.

**Figure 10.**
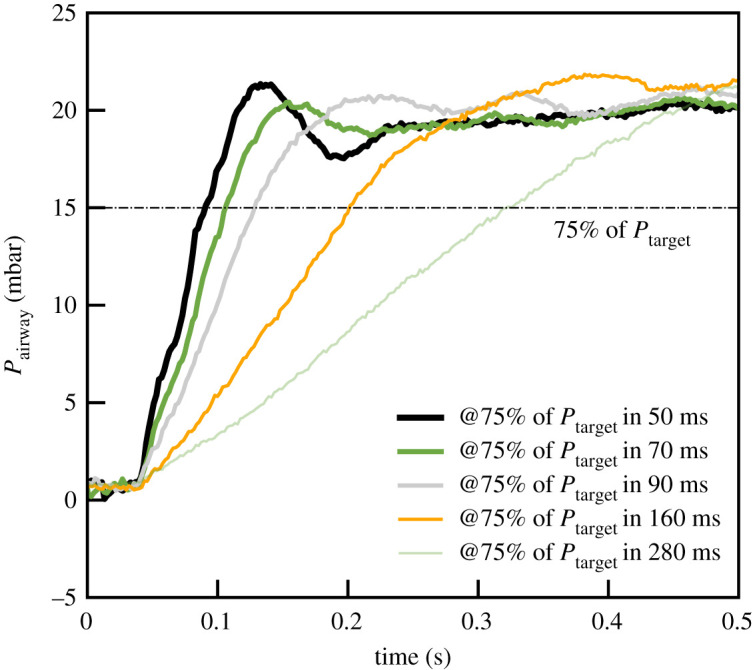
Rising edge of the inhalation for various rise time settings. The lung compliance here is 50 ml cm^−1^ H_2_O for a resistance of 5 cm H_2_O l^−1^ s^−1^.

Thanks to the flexibility provided by the PID control, the time needed to reach the maximum of pressurization can be tuned between the fastest setting of about 50 ms up to about 300 ms, as illustrated in figure 10. The tuning of the PID parameters can be challenging due to the wide variety of patient conditions ranging from patients with high flow resistance such as COPD to low compliance such as in acute respiratory distress syndrome (ARDS) patients. A set of parameters were chosen to optimize the PID controller for a range of patient profiles. In a final implementation, it should be possible for the algorithm to detect situations where the performance is non-optimal for an individual patient. For instance, too much ringing on the plateau pressure caused by a very resistive patient could cause the software to suggest to the clinician an adjustment of the parameters, such as moving to a slower rise time setting.

**Figure 11.**
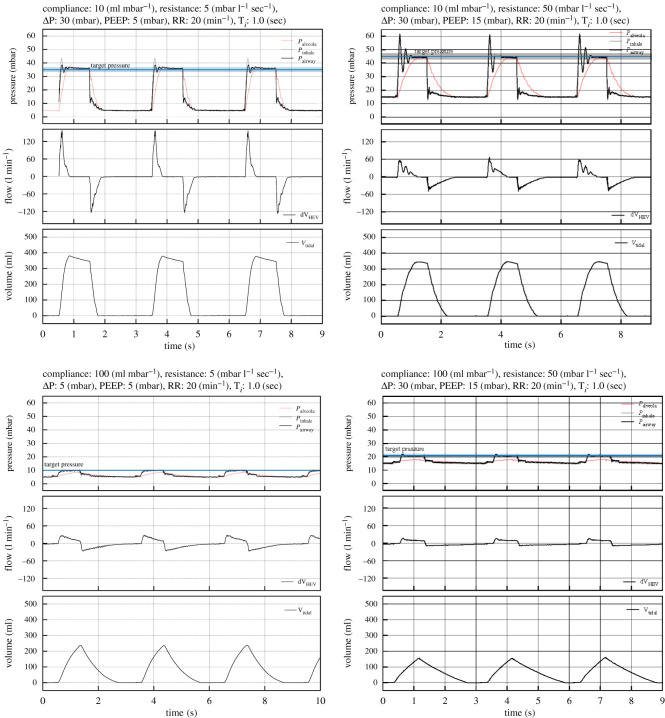
Pressure, flow and volume registered for four different patient configurations to illustrate the performance of the ventilator with the lowest and highest lung compliance, together with the lowest and highest airway resistance. The ventilator copes correctly with all conditions, showing, as expected, a more peaked flow, as the compliance and resistance is reduced.

The response of the HEV ventilator to lungs with compliance varying from 10 to 100 ml H_2_O and with resistance from 5 to 5 cm H_2_O l^−1^ s^−1^ has been studied. The PEEP values range from 5 to 15 cm H_2_O and target pressure up to 45 cm H_2_O were tested. A sub-sample of the pressure, flow and volume curves are reported in figure 11. All data were taken with the same settings of the controller, favouring fast rise times. These settings cause oscillations for highly resistive lungs, which are not present with settings of normal or slow rise times, as can also be seen in figure 10; in practice, this parameter could be adjusted automatically or by a choice on the touchscreen. As expected, higher resistances naturally slow down the rise of the inspiratory pressure (at the airways). Lung compliances lower than 10 cm H_2_O ml^−1^ could not be tested with this lung simulator but will be the subject of a dedicated test later; extrapolating from the current results, we expect a consistently good performance.

### 5.2. Volume calculation

**Figure 12.**
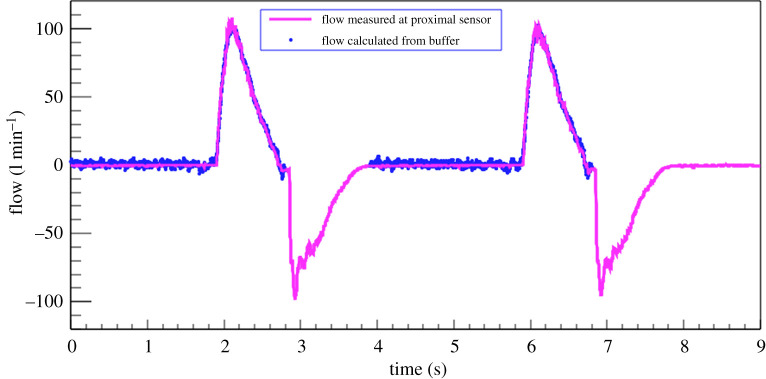
Volume during the inhale phase as calculated from the pressure drops in the buffer, compared with the value measured by the proximal flow sensor.

Thanks to the fact that the air is always supplied via a buffer, for which the parameters are well measured, HEV is able to calculate the volume of air delivered to the patient independently of the proximal flow sensors. This can provide a valuable second source of information in case the sensors become faulty or blocked by humidity, and is considered to be an additional safety feature of the HEV. The HEV flow measurement with the Hamilton differential pressure sensor was calibrated using a Sensirion flow sensor bridge.^3^ To ensure accuracy the sensor bridge measurements were compared with a Vögtlin Instruments red-eye high precision thermal mass flow sensor;^4^ excellent agreement was found. The comparison between the buffer calculation and the flow sensor calculation in a typical breathing cycle is shown in figure 12. It can be seen that the buffer calculation accurately estimates the volume of air leaving the buffer throughout the entire inhale cycle.

### 5.3. Inhale trigger performance

The inhale and exhale triggers are essential to guarantee the comfort of the patients and to ensure fast recovery time. The trigger functionalities were developed with this aspect in mind and particular effort was made to qualify them.

#### 5.3.1. Inhale trigger algorithm description

Whenever the patient initiates a breath, the proximal flow sensor detects an increased flow and a drop in pressure. The increase in flow is used as a trigger for the inhale sequence. The inhale trigger algorithm works in the following way. Whenever the flow reaches 10% of the maximum exhale flow, the window allowing for an inhale trigger is opened. This condition is by definition always met when the patient initiates an inhalation. The expected flow at a given instant is computed by a linear regression algorithm from a given time window before that point. It provides the baseline, with which the measured flow is compared. If the measured flow, corrected for the baseline, is above a threshold that can range from 0.2 to 20 l min^−1^, then the inhalation starts. Lower thresholds are sensitive to noise, in particular that induced by the heart–lung interactions. This threshold is set by the clinician. Other means of triggering such as a pressure-based trigger or a combination of flow and pressure can be easily incorporated using the modular software design.

#### 5.3.2. Inhale trigger qualification

**Figure 13.**
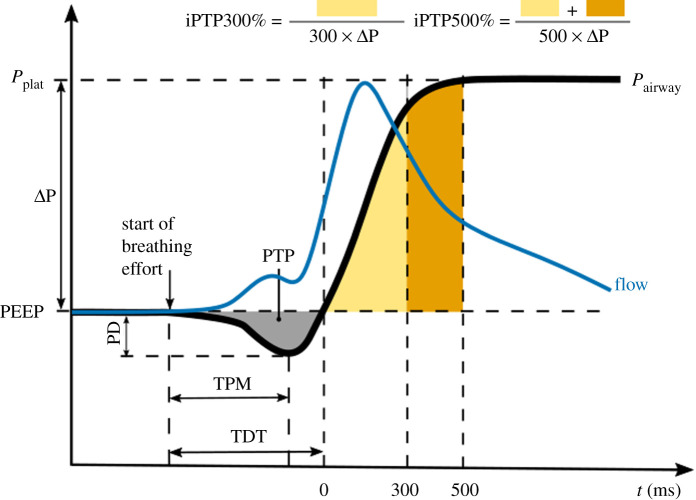
Measured parameters to qualify the inhalation trigger. The variables used in this work are based on the work presented in [31,33,37].

To qualify the performance of the inhale trigger, the variables defined in [31], which are based on the parameters originally defined in [32] are used. Figure 13 illustrates schematically the typical behaviour. The beginning of the inhalation effort is directly taken as the signal from the TestChest when breathing effort is about to start, typically a little time before a visible pressure drop. The flow can be seen to rise, triggering the HEV and soon after a steep pressure drop develops, followed by the rise of the pressurization curve. The following variables are defined: the time to minimum pressure (TPM) is defined as the time between the beginning of the inhalation effort and the minimum value measured by the proximal flow sensor, and the trigger delay time (TDT) is defined as the time between the beginning of the inhalation effort and the moment the pressure returns to zero. Values of TPM in commercial ventilators typically vary between 50 and 150 ms depending on the inhalation effort (see for example [31]), while TDT, which should ideally be below 150 ms so as not to be felt by the patient, varies for commercial ventilators in practice between 90 and 250 ms [31]. The pressure-time product during trigger (PTP) is represented in figure 13 by the yellow/brown areas and represents the effort until the pressure is effective. It ranges from 0.02 to 0.3 cm H_2_O s in commercial ventilators [31]. The ideal iPTP300% (iPTP500%) percentage is the ratio of the pressure integral over the 300 (500) ms following the trigger delay (the area of the yellow/brown regions in figure 13) and the ideal PTP at 300 (500) ms. It should be as large as possible, with typical commercial ventilators exhibiting values between 10 and 50% (20 and 75%) for PTP300 (PTP500) depending on the inhalation effort [31].

To test the inhalation trigger, the same lung parameters, pressurization parameters and inhalatory effort as in [31] were used. TestChest was set with a compliance of 50 cm H_2_O ml^−1^ and a resistance of 5 cm H_2_O l^−1^ s^−1^, and breath cycles were set at 12 respirations per minute. The breaths consist of a 1 s inhalation with a reduction of inspiratory pressure at a constant rate, with an occlusion pressure at 100 ms of (*P*_0.1_) of 2 cm H_2_O for low effort breaths and 4 cm H_2_O for high effort breaths. Several pressurization parameters were used: ΔP of 10, 15 20 and 25 cm H_2_O each tested with a PEEP of 0 and 5 cm H_2_O. The inhale trigger threshold was set to 0.5 l min^−1^.

Table 1 summarizes the results of the inhale trigger qualification. The results of the two PEEP values are within errors such that they are averaged in the table. Comparing the results with the ventilators studied in [31], the HEV inhale trigger appears to perform very well.

**Table 1.** Results of the inhale trigger characterization. The results are averaged over a minute of breathing (respiratory rate of 18 per minute) with a PEEP of 0 cm H_2_O. The typical measurement error of each variable is reported next to the variable name.

	small effort				large effort			
	*P*_0.1_ = 2 [cm H_2_O]				*P*_0.1_ = 4 [cm H_2_O]			
Δ*P* [cm H_2_O]	10	15	20	25	10	15	20	25
TPM [ms] ±7 ms	105	107	108	111	104	105	106	108
TDT [ms] ±5 ms	131	123	122	123	149	126	119	119
PD [cm H_2_O] ±0.2 cm H_2_O	2.7	2.5	2.5	2.6	4.3	4.3	4.2	4.4
PTP [cm H_2_O s] ±0.01 cm H_2_O s	0.11	0.09	0.09	0.08	0.20	0.17	0.15	0.15
PTP300 [%]±2%	67	65	67	64	61	61	61	67
PTP500 [%]±1%	81	77	78	74	79	77	76	80

The trigger response was studied again in the presence of leaks with PEEP settings at $0\;\mathrm{cm}\;H_{2}O$ and $\Delta P=20\;\mathrm{cm}\;H_{2}O$. Four settings were examined: no leaks, weak, medium and strong leaks as set by TestChest. All results agree with the previously measured values within 10%.

**Figure 14.**
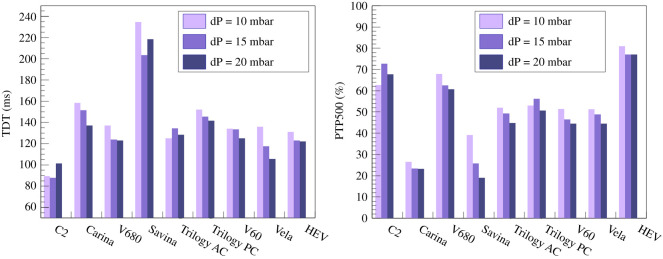
Measured parameters compared with similar published measurements for commercial ventilators [31]. Since the conditions cannot be guaranteed to be precisely similar, the most conservative definitions possible are taken for this comparison. The HEV can be seen to perform at a similar level to the commercial devices.

The HEV measured parameters may be compared with similar published measurements for commercial ventilators [31]. Since the conditions may not be precisely identical, the most conservative definitions possible are taken for this comparison; in particular, the HEV reaction time is measured from the moment that the electrical inhalation signal is triggered in the TestChest. As an example, the TDT and PTP500 values are displayed in figure 14. The HEV can be seen to perform towards the top of the range of the commercial devices for both these parameters.

### 5.4. Oxygen mixing test

The O_2_ concentration in the buffer, typically characterized as FIO_2_, or fraction of inspired oxygen, is controlled by taking advantage of the fact that during the regular buffer refill phase it is completely isolated from the patient. If a sharp increase in oxygen is demanded, the buffer can at this moment be depressurized to atmospheric pressure and then filled with pure O_2_. Over the course of a number of breaths the O_2_ concentration reaches the target value, after which it is maintained by refilling with air and O_2_ sequentially. The reverse procedure is followed to reduce the O_2_ concentration. A needle valve attached to the tank allows a small leakage towards the oxygen sensor which is therefore isolated from the medical air pathway, giving flexibility and cost optimization in the choice of this sensor. An advantage of this method is that the O_2_ concentration is precisely known by calculation, and the sensor acts to confirm the expected value. The sensors used are based on zirconium dioxide and are accurate (0.5% O_2_), fast (response time less than 4 s), inexpensive and readily available.

**Figure 15.**
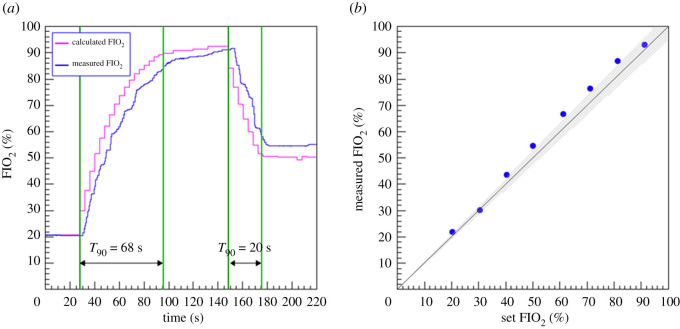
(*a*) Measured FIO_2_ as function of the expected O_2_ percentage as calculated from the relative time from the initial demand. (*b*) Achieved versus set O_2_ percentage. The grey band represents the region within ±5% of the set value.

Figure 15 shows a test cycle where the oxygen concentration demanded is first raised to 95%, held for a short time, and then decreased to 50%. The respiratory rate was set to 15 breaths per minute. The calculated value is shown as well as the measured value. In both cases, the time taken to reach 90% of the target value is shown, taking as reference the measured value, which lags somewhat behind the calculated value. The measured FIO_2_ as function of the expected O_2_ percentage as calculated from the pressure change inside the buffer is shown on the right of the figure. The measured FIO_2_ in the lung is within 5% of the set value, which is an acceptable performance.

## 6. Conclusion

HEV has been developed to be a high-quality, low-cost ventilator, suitable for use in a hospital setting, capable of providing pressure and volume control modes, pressure support, CPAP and oxygen-enriched air. The design is intended for easy and fast manufacturing and can be performed in a decentralized way with affordable and readily available parts. The central concept of the design with a gas accumulator gives many advantages in terms of robustness, safety, affordability and precise pressurization behaviour. The electrical design is conceived in a modular way for quick prototyping and deployment, which facilitates mass production. HEV is intended to be robust and adaptable for a wide range of geographical deployment, including in regions where compressed air may not be readily available and an alternative air source can be used. The results presented in this article demonstrate quantitatively the pressurization, triggering and oxygen mixing performance of the HEV.

The final production of a ventilator is challenging. HEV has been designed according to all relevant norms and with mandatory safety features inherently built in. The limitations of the HEV prototypes must be addressed in a final version, in particular, a manual PEEP was used for the prototypes which must be replaced by a controlled valve to match the high-quality specifications of the rest of the device.

HEV has been externally tested at the ETH Zurich Chair of Product Development and Engineering Design Ventilator test rig. In pressure control mode HEV accurately achieves the target pressures, with fast rise time which is adjustable to slower times on clinician request. Special attention has been paid to the inhale and exhale triggers to optimize patient comfort. The inhale trigger, based on the flow measurement, accurately reacts to the patient effort, with short rise times and excellent PTP values. The system displays and monitoring use concepts familiar to particle physics such as the possibility for remote monitoring from screens or mobile devices, data logging for quality control and performance monitoring, and remote training, with the possibility for appropriate additional security measures for use in a clinical setting. Several working prototype devices have been installed at HEV institutes external to CERN, among others at Universidade Federal do Rio de Janeiro (UFRJ), Benèmerita Universidad Autónoma de Puebla (BUAP) and at the National Technical University of Athens (NTUA), as well as the Fraunhofer Research Institution for Individualized and Cell-based Medical Engineering (IMTE), Lübeck. These prototypes are being used to carry out additional developments such as performance testing, development of an independent compressed air supply, development of calibration methods, and implementation of code to calculate and display the volume flux circulating in every breathing cycle. Additionally, these devices are used as research demonstrators for advanced control algorithms and automated support systems.

As far as production is concerned, it is foreseen, on the one hand, to enable this through providing partner academic institutions with the detailed design for these institutions to follow up in accordance with local possibilities and standards; on the other hand, directly through industry, non-governmental, governmental and international organizations, such as the World Health Organization (WHO), for which purpose discussions are ongoing and contacts have been established with potential partners. HEV has been selected by WHO for inclusion in the 2021 Compendium of Innovative Health Technologies [34]. A consortium based in the UK, the HPLV collaboration, funded by a UKRI GCRF grant [35], is currently addressing the need to establish an accessible hardware and software platform which can be used by manufacturers to more easily achieve medical certification of a device based on the HEV, focusing on OECD-DAC countries. HEV licences have been signed or are under discussion with various partners, notably a company based in Switzerland^5^ and a company in India^6^ which is planning to adapt the package to address local needs.

Every effort is being made to finalize the design of the HEV in accordance with the state-of-the-art best practices and standards. It is intended that the formal certification process must be initiated by the parties that decide to place this device on the market. The hardware and software design has been done in a flexible way which allows the development of different modes of operation, for instance volume control modes which in principle can be developed and applied as a firmware update. In addition, the HEV prototypes can be used as a testbench to quickly implement and test novel algorithms or hardware updates, and in this way could provide a fresh avenue for medical research.

## Data Availability

This article has no additional data

## Footnotes

1 European Council for Nuclear Research, https://home.web.cern.ch.

2 See https://hev.web.cern.ch.

3 SEK-SFM3019

4 reftofill

5 Jean Gallay SA.

6 AXIS enterprises PVT. Ltd.
